# The Epidemiological Characteristics of Epilepsy in the Province of Khyber Pakhtunkhwa, Pakistan

**DOI:** 10.3389/fneur.2018.00845

**Published:** 2018-11-06

**Authors:** Shakir Ullah, Niaz Ali, Adnan Khan, Saad Ali, Haleema Rehana Nazish

**Affiliations:** ^1^Pharmacology/Institute of Basic Medical Sciences, Khyber Medical University, Peshawar, Pakistan; ^2^Center for Neuroscience, Shantou University Medical College, Shantou, China; ^3^Neurology Departments, Lady Reading Hospital Peshawar, Peshawar, Pakistan

**Keywords:** epilepsy, demographics, clinical outcome, social profile, socioeconomic status, types of seizures, carbamazepine, valproic acid

## Abstract

Previous studies have shown that Khyber Pakhtunkhwa, Pakistan has a high incidence of epilepsy and a high proportion of low socioeconomic background and high treatment gap. Considering the changes over the past 20 years little is known about the current epidemiological characteristics of epilepsy in Khyber Pakhtunkhwa, Pakistan. The current study was focused to find the impact of various contributing factors on the clinical response to anti-epileptic drugs in the KP population, Pakistan. A total of 315 participants aged 19.1 ± 8.6 years were examined. Mean age of the patients was 18 ± 8.1 year. Epilepsy was high in male patients (64.39%) and urban areas (60.1%). Mostly, 88.6% of patients were belonging to low socioeconomic status background. 42.4% patients have positive family history for epilepsy and 42.8% patients had consanguineous marriages. Middle SES class patients (OR, 2.22 [CI, 0.54–9.1]) were slightly associated with controlled response to CBZ and VPA therapy. Absence seizure (OR, 1.16 [CI, 0.59–2.3]), and Complex partial seizure (OR, 1.29 [CI, 0.58–6.3]) showed good response to CBZ therapy while, Myoclonic seizure (OR, 2.23 [CI, 0.05–8.8]) was responsive to VPA therapy. However, non-compliance (*R*^2^ 0.82, *P* < 0.0001) and nature of seizures (*R*^2^ 0.83, *P* < 0.0001) were associated with the high risk for poor response to both CBZ and VPA therapy. Epilepsy was high in male patients and in urban areas. Most patients were belonging to low socioeconomic status. Non-compliance, low socioeconomic and nature of seizures strongly predict poor clinical response of anti-epileptic drugs therapy.

## Introduction

Epilepsy is a neurological disease, which is characterized by recurrent seizures that may occur suddenly without any provoking factors (1). Approximately 70 million people are suffering from epilepsies throughout the world. Epilepsy contributes 1% burden to global diseases while this contribution is 80% in the developing countries (2). Both developing and developed countries have different health-care priorities according to the need for primary prevention, to the recognition of seizures, and access to sustainable and appropriate therapy. Different approaches are used to properly treat epilepsy in the context of regional resources depend on the prevalence of a disease. Diversities in demographic factors such as gender (3), race (4), age (3), geographic area (5), religion, and culture (5) along with provoking factors are associated with poor responsive epilepsy.

Demographic and clinical factors have a crucial role in the management of epilepsy. False believes, low SES have a very important role in the poor management of epilepsy. Similarly, consanguineous marriages among the positive family history for epilepsy can increase the burden of epilepsy in a society. It is well-known that the incidence of epilepsy is linked to low socioeconomic status, positive family histories of epilepsy, limited access to health care system and environmental factors (6). Reports suggest that socioeconomically deprived people are more susceptible to epilepsy. The association is strongly supported by the facts that the incidence of epilepsy is high in developing countries as compared to developed countries (7). Several other studies also corroborate that socioeconomic status (SES), positive family history and poor compliance has an established association with resistant epilepsy (8–10). Health related quality of life (HRQL) is associated to proper response to AEDs and it has been reported that clinical response may vary across epileptic patients with different clinical, demographic, and socioeconomic variations (11, 12). With regard to clinical variables, seizures types and frequency have been found to be significant predictors of HRQL scores and clinical response (13–5). It has been found that psycho-social factors is also related to quality of life (QOL) in Korean epilepsy patients. Good QOL is directly related to better clinical response to AEDs and recognition of these factors will lead health professionals to develop different strategies to improve the QOL of these patients (15). Common clinical factors, seizure frequency, is closely associated with poor outcomes, QOL, and mortality (16). As these above variables are associated with epilepsy. Therefore, it has been suggested that demographic factors, gender difference, culture aspects, beliefs in different religion, geographic variations, and SES of an patients with epilepsy may also affect clinical response of a drug and will widen treatment gap. These are the factors in which interventions are very easy and treatment response can be improved which can reduce the treatment gap. Therefore, the purpose of the present study is used to examine the impact of these factors on the clinical outcomes of CBZ and VPA therapy in epileptic in Pakhtun population of Khyber Pakhtunkhwa, Pakistan. It will help us to improve the clinical response to carbamazepine and valproic acid therapy by exploring the impact of demographic and social profile of the patients during treatment.

## Materials and methods

### Study design and protocol

**Figure 1:**
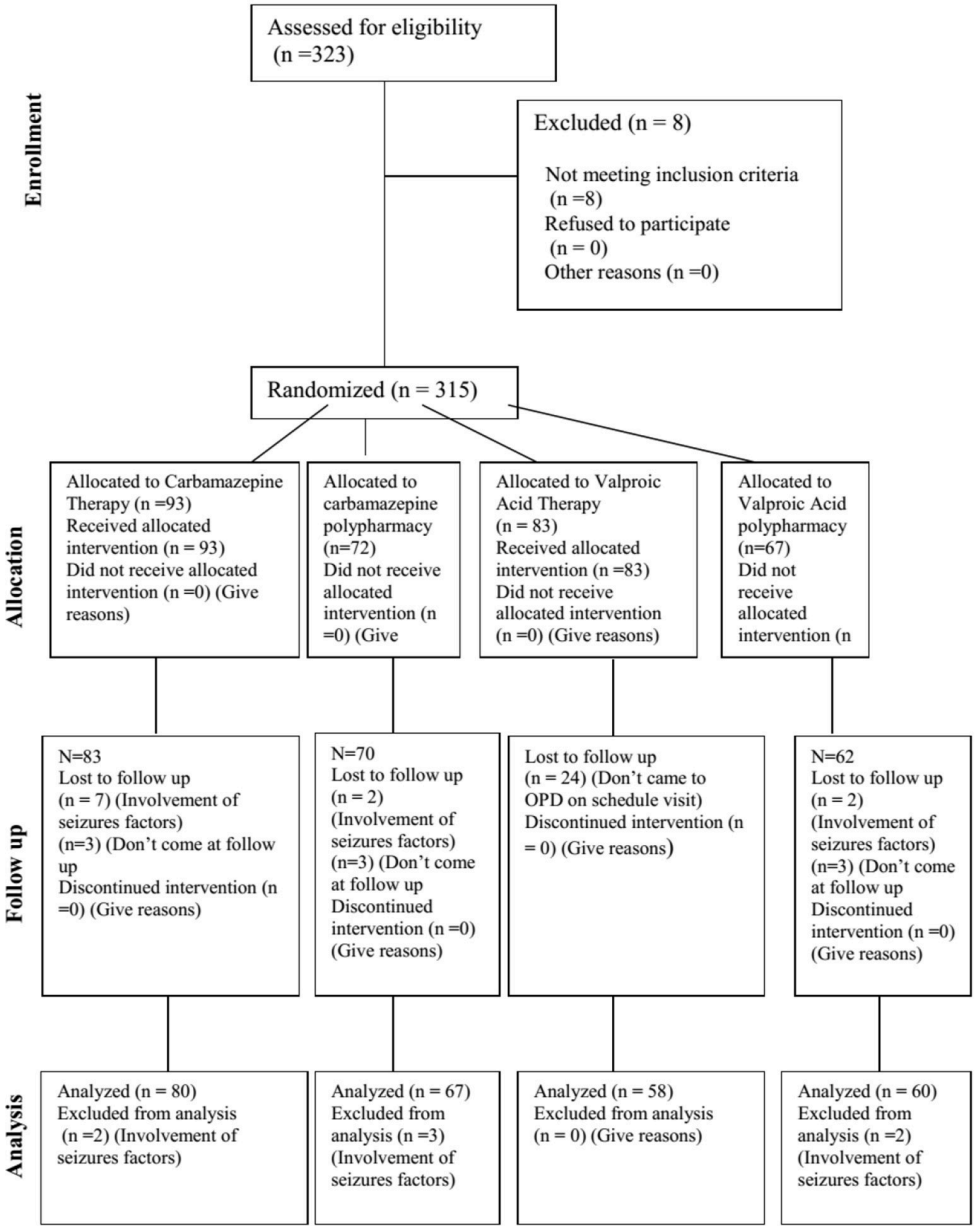
CONSORT diagram. CONSORT diagram showing the flow of participants through each stage of a randomized trial.

Patients with epilepsy sample size (*N* = 323) was calculated at 95% CI with SD of 8 and marginal error of 1. Keeping in view 20% drop out total patients included in the study were 315. Patients from Khyber Pakhtunkhwa, Pakistan who visited the Neurology Department of Government Lady Reading Hospital for opting treatment were included in the study on consent, assent, and surrogate assent form. The study was started in August 2014 and completed in April, 2016. The study was executed according to the CONSORT flow chart presented in Figure 1.

Ethics Board of the Khyber Medical University, Peshawar approved the study via no: DIR/KMU-EB/AC/000047 dated 04/07/2014. The study was conducted according to Helsinki declarations. The data was collected on a standard questionnaire designed according to the protocol of the WHO which includes all those necessary information to reduce the biases in the study.

Mean dose of carbamazepine was 452 mg/day and valproic acid 859 mg/day and was titrated for better management of epilepsy at scheduled visits.

### Inclusion criteria

Patients with epilepsy who were suitable candidate for carbamazepine and valproic acid as monotherapy or polypharmacy for first time or newly diagnosed for epilepsy were enrolled in the study. Patients with epilepsy who were willing to participate in the study after explaining the steps and the aim of the study in the context of local language were included in the study upon consent and surrogate consent form. Seizures provocative factors were controlled by proper counselling of the patients and ensure proper clinical response to CBZ and VPA as monotherapy or polypharmacy.

### Exclusion criteria

Patients with epilepsy were not included in the study who was not suitable candidate for carbamazepine and valproic acid as monotherapy or polypharmacy. Patients were also excluded from the study that was not willing to participate in the study or were suffering from some other co-morbidity. Non-compliant patients were also excluded from the study.

### Assessments of seizure characteristics and socioeconomic status

Types of epilepsy were diagnosed according to guidelines of International League against Epilepsy (ILAE), witness presentation; family history; EEG report and expert opinion of concern OPD/Ward neurologists. All available information was considered in the classifications of seizures, including the medical record, interviews, EEG, and brain imaging. The SES of patients was are classified as reported by Association American Psychology report 2011 (17). The higher Socioeconomic class will have 50–100 and above, Middle class 15–49, and Low economic class will have below 15 Per capita monthly income limits (Rs.) (17).

### Measurement of seizure control in patients with epilepsy

Seizures' control was recorded in the form of reduction of frequencies of seizures using standardized proforma in local language. The numbers of seizures per week were recorded at baseline and these patients were then evaluated for seizure control at 3rd and 6th month of the therapy. The patients with epilepsy compliance to the medication were checked by counting the pills remaining in the strip on scheduled visits. Drug response was determined as either freedom from seizures or reduction in number of seizure per week after initiation of carbamazepine and valproic acid therapy at 3rd and 6th month.

### Statistical analysis

The distribution of age, clinical, and demographic characteristic of epileptic patients were presented in the form of percentage. The Mean ± SD were determined for age. The expected outcome (EXP B) was determined using univariate logistic regression analysis. The prediction of clinical response with respect to all variables was determined using multivariate regression analysis by incorporating step by step variables and to determine the combined as well as individual impact of each variable on clinical outcomes of CBZ and VPA as monotherapy or polypharmacy. SPSS 20 and Stata version 12 was used for analysis.

## Results

### Demographic features of epileptic patients

Mean age of epileptic patients were 19.1 ± 8.6 years (range 1–42) (Table 1). The prevalence of epilepsy was higher in patients having age of 10–20 years. Epilepsy was high in male (64.39%) than female (35.98%) patients in study population (Table 2). The percentage of cousin marriage and positive family history of epilepsy was (42.8%) and (42.4%) (Table 2). The level of education was very low in these patients. Furthermore, these patients were having low socioeconomic status (SES) (88.63%).

**Table 1:** Age and gender wise distribution of epilepsy in 315 epileptic patients.

**Age range (years)**	**Total Number, *n* (%)**	**Mean age ±SD**	**Male, *n* (%)**	**Female, *n* (%)**
1–10	47(14.9)	7.3 ± 2.5	22 (46.8)	25 (53.2)
11–20	165 (52.4)	16.1 ± 2.8	102 (61.8)	63 (38.2)
21–30	69 (22.7)	24.3 ± 2.6	45 (75.0)	11 (25.0)
31–40	31 (21.9)	33.5 ± 3.0	21 (67.7)	10 (32.3)
41–50	3 (0.95)	46.3 ± 5.1	2 (66.7)	1 (33.3)
Total	315	19.1 ± 8.6	200 (63.5)	115 (36.5)

**Table 2:** Demographic and clinical features of epileptic patients.

**Demographic data**	**Patients (*N* = 315), *n* (%)**
Male	200 (63.5%)
Female	115 (36.5%)
Urban	181 (57.5%)
Rural	134 (42.5%)
Cousin marriages	148 (46.9%)
Non cousin marriage	167 (53.1%)
Family history Positive	135 (42.9%)
Family history Negative	180 (57.1%)
Employee	22 (6.9%)
Labor	293 (93.1%)
Know about epilepsy	239 (75.9%)
Don't Know about epilepsy	76 (24.1%)
Educated Patients (Middle grade)*	41 (13.0%)
Uneducated patients	274 (87.0%)
Low SES	261 (82.7%)
Middle SES	54 (17.3%)
1-Pre-school system (3–5 Years)
2-Primary (Grade 1–5)
3-Middle (Grade 6–8)
4-High (Grade 9–10)
5-Intermediate (Grade 11–12)

* *Grading system of educational level*.

### Phenomenology

**Figure 2:**
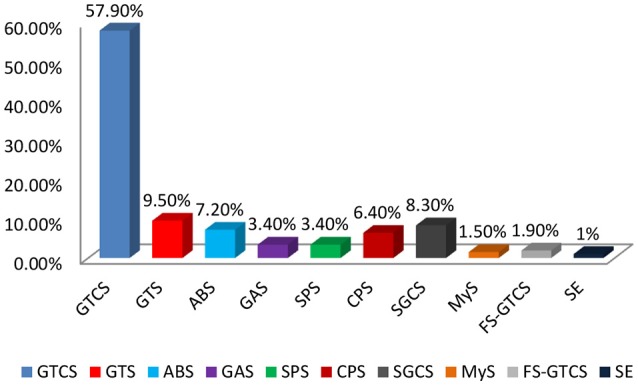
Types of seizures in Pakhtun population.

Types of seizures were classified according to the ILAE guidelines and clinical presentation of patients with epilepsy. Generalized tonic clonic seizures was commonly observed 153 (57.9%) during this study in epileptic patients of KP (Figure 2). Other types of seizures were Generalized tonic seizures 25 (9.5%), Absence seizures 19 (7.2%), Generalized atonic 8 (3.4%) secondary generalized complex seizures 22 (8.3%), simple partial seizures 9 (3.4%), and complex partial seizures 17 (6.4%) were found in the target population (Figure 2).

### Impact of demographic and clinical features vs. response of anti-epileptic drugs

The association of different demographic variables with clinical outcome were determined using univariate logistic regression analysis (Table 3). It has been found that high education level were slightly associated with controlled response (OR, 1.84 [CI, 0.50–6.7]) to CBZ and VPA as monotherapy or polypharmacy. Similarly, good SES was also associated with good response (OR, 2.22 [CI, 0.54–9.1]) to CBZ and VPA as monotherapy or polypharmacy. Absence seizure (OR, 1.16 [CI, 0.59–2.3]), Complex partial seizure (OR, 1.29 [CI, 0.58–6.3]), and Myoclonic seizure (OR, 2.23 [CI, 0.05–8.8]) showed a good response to CBZ and VPA as monotherapy or polypharmacy (Table 3).

**Table 3:** Impact of different factors on clinical response of CBZ and VPA therapy.

**Variables**	**Exp (B) or (OR)**	**CI (95%)**
Gender	1.18	0.63–2.2
Area	0.67	0.36–1.2
Cousingenous marriages	0.91	0.50–1.6
Family history	0.84	0.45–1.5
Perception	0.69	0.32–1.4
Education	1.84	0.50–6.7
SES	2.22	0.54–9.1
Compliance	0.66	0.32–1.3
Generalized tonic clonic seizure	0.58	0.03–10.0
Generalized tonic seizure	0.98	0.04–20.0
Absence seizure	1.16	0.59–2.3
Generalized atonic seizures	0.81	0.02–6.8
Simple partial seizure	0.44	0.02–11.5
Complex partial seizure	1.29	0.58–6.3
Secondary generalized complex seizure	0.22	0.01–4.9
Myoclonic seizure	2.23	0.05–8.8
Febrile seizure	0.29	0.08–10.1
Status Epilepticus	0.31	0.25–1.8

*The reference category is poor controlled seizure, EXP (B) mean expected value of prediction using univariate regression analysis*.

Similarly, multivariate regression models were applied to demographic variables to predict clinical response of CBZ and VPA as monotherapy or polypharmacy in epileptic patients. Over all the factors added in Model 9 (*R*^2^ 0.82, *P* < 0.0001) and model 10 (*R*^2^ 0.83, *P* < 0.0001) were found to increase the risk of poor response to AEDs.

## Discussion

In our study the prevalence rate of epilepsies was high in male patients where most of them were belonging to urban areas. Our observations were in line to Khan et al. (18) that epilepsy was high in male population and urban areas (18). Though we found an inverse figure as reported by Mohammad et al. where epilepsy is more prevalent in female than male patients in Iran (19). We found that epilepsy was common in patients having age group of 10–20 years. These findings were in line with the reports of previous studies conducted in Pakistan and India (20–26). The percentage of consanguineous marriages and positive family history of epilepsy was high in the study population. The SES of most patients observed in our study was very low. Heaney et al. observed that low SES has an association with epilepsy because of low quality of life increased the susceptibility of an individuals to different provoking factors of epilepsy (27). Other studies also show that low SES was associated with an increased risk for epilepsy in individuals with seizures of unknown etiology (28–35). Luengo et al. reported that genetic history of patients with epilepsy have a 2.5 times higher risk for epilepsy in their coming generation (36). GTCS was common among other types of seizures in the target population which is in line with Nowshad et al. observations (18). It has been found that clinical outcome in patients with absence seizures and myoclonic seizures were good compared to other types of seizure. Poor response was also high in non-compliance patients. Regression model showed that poor compliance and nature of seizures significantly associated with poor response to CBZ and VPA as monotherapy or polypharmacy even at optimal adjusted dose of CBZ and VPA as monotherapy or polypharmacy. Thomson et al. (38) stat that compliance has a direct association with seizure response (37, 38). Dragoumi et al. (39) demonstrate that types of seizure also affect clinical outcome in epileptic patients (39). If patients show non-compliance with therapy and do not take their AEDs according to prescription then it leads to poor clinical outcome. Similarly, low SES of patient is also a hurdle in proper compliance and lead to poor clinical response. Low SES also responsible for large treatment gap. Nature of seizure is also a main factor for resistant epilepsy.

## Limitations and strength of the study

It is a hospital based study which doesn't show the clear picture of whole society. It has an advantage that this study will provide the platform to conduct a community based study to determine the prevalence and stigmatization of epilepsy.

## Conclusion

Male patients were having a high prevalence (64.39%) of epilepsies where 60.1% were from urban areas. Epilepsy was high in patients with age ranging 11–20 years. Positive family history of epilepsy and consanguineous marriages was the key characteristics of the epileptic patients. Non-compliance and nature of seizures strongly predict poor clinical response of Anti-epileptic drugs therapy.

## Availability of data and material

The analyzed dataset during the current study will be provided from the corresponding author on reasonable request.

## Author contributions

SU carried out experimental work as Ph.D. Scholar. Also prepared the 1st draft of manuscript. NA extensively revised the manuscript. He is also designed the study and wrote the research project. AK helped in diagnosis and patients follow up for their seizures control. SA helped in Clinical scoring of epilepsies. HN helped in experimental work. All authors approved the final version of manuscript.

### Conflict of interest statement

The authors declare that the research was conducted in the absence of any commercial or financial relationships that could be construed as a potential conflict of interest.

## Abbreviations

AEDs: Anti-epileptic drugs
CBZ: Carbamazepine
HRQL: Health related quality of life
ILAE: International League against Epilepsy
QOL: quality of life
SES: Socioeconomic Status
VPA: Valproic Acid.

## References

[1] Brodie M Engel J Lee P de Boer H European white paper on epilepsy-Call to action. EPILEPSIA (2003) 44:4–4. 12919341

[2] World Health Organization Atlas: Epilepsy Care in the World (2005).

[3] Ernst MM Johnson MC Stark LJ. Developmental and psychosocial issues in cystic fibrosis. Child Adolesc Psychiatr Clin N Am. (2010) 19:263–83. 10.1016/j.chc.2010.01.004 20478499 PMC2874200

[4] Barr HL Britton J Smyth AR Fogarty AW. Association between socioeconomic status, sex, and age at death from cystic fibrosis in England and Wales (1959 to 2008): cross sectional study. BMJ (2011) 343:d4662. 10.1136/bmj.d4662 21862532 PMC3160750

[5] Djibuti M Shakarishvili R. Influence of clinical, demographic, and socioeconomic variables on quality of life in patients with epilepsy: findings from Georgian study. J Neurol Neurosurg Psychiatry (2003) 74:570–3. 10.1136/jnnp.74.5.570 12700294 PMC1738431

[6] World Health Organization Epilepsy: aetiology, epidemiology, and prognosis. In: Epilepsy: Aetiology, Epidemiology, and Prognosis. World Health Organization (2001) p. 4.

[7] Neligan A Hauser W Sander J. The epidemiology of the epilepsies. Handb Clin Neurol. (2012) 107:113. 10.1016/B978-0-444-52898-8.00006-9 22938966

[8] Birbeck G Chomba E Atadzhanov M Mbewe E Haworth A. The social and economic impact of epilepsy in Zambia: a cross-sectional study. Lancet Neurol. (2007) 6:39–44. 10.1016/S1474-4422(06)70629-9 17166800 PMC2938018

[9] Kelvin EA Hesdorffer DC Bagiella E Andrews H Pedley TA Shih TT. Prevalence of self-reported epilepsy in a multiracial and multiethnic community in New York City. Epilepsy Res. (2007) 77:141–50. 10.1016/j.eplepsyres.2007.09.012 18023147

[10] Noronha AL Borges MA Marques LH Zanetta DM Fernandes PT De Boer H. Prevalence and pattern of epilepsy treatment in different socioeconomic classes in Brazil. Epilepsia (2007) 48:880–5. 10.1111/j.1528-1167.2006.00974.x 17326788

[11] Baker GA Jacoby A Buck D Brooks J Potts P Chadwick DW. The quality of life of older people with epilepsy: findings from a UK community study. Seizure (2001) 10:92–9. 10.1016/S1059-1311(00)90465-5 11407951

[12] Suurmeijer TP Reuvekamp MF Aldenkamp BP. Social functioning, psychological functioning, and quality of life in epilepsy. Epilepsia (2001) 42:1160–8. 10.1046/j.1528-1157.2001.37000.x 11580765

[13] Baker GA Gagnon D McNulty P. The relationship between seizure frequency, seizure type and quality of life: findings from three European countries. Epilepsy Res. (1998) 30:231–40. 10.1016/S0920-1211(98)00010-2 9657650

[14] Berto P. Quality of life in patients with epilepsy and impact of treatments. Pharmacoeconomics (2002) 20:1039–59. 10.2165/00019053-200220150-00002 12456200

[15] Choi-Kwon S Chung C Kim H Lee S Yoon S Kho H. Factors affecting the quality of life in patients with epilepsy in Seoul, South Korea. Acta Neurol Scand. (2003) 108:428–34. 10.1046/j.1600-0404.2003.00151.x 14616296

[16] Baker GA Nashef L Hout BA. Current issues in the management of epilepsy: the impact of frequent seizures on cost of illness, quality of life, and mortality. Epilepsia (1997) 38:S1–8. 10.1111/j.1528-1157.1997.tb04511.x 9092951

[17] AP Association Education and Socioeconomic Status (2011).

[18] Khan N Jehan B Khan A Khan H Audit of 100 cases of epilepsy in a tertiary care hospital. Gomal J Med Sci. (2011), 42–5.

[19] Mohammadi MR Ghanizadeh A Davidian H Mohammadi M Norouzian M. Prevalence of epilepsy and comorbidity of psychiatric disorders in Iran. Seizure (2006) 15:476–82. 10.1016/j.seizure.2006.05.011 16931061

[20] Aziz H Güvener A Akhtar S Hasan K. Comparative epidemiology of epilepsy in Pakistan and Turkey: population-based studies using identical protocols. Epilepsia (1997) 38:716–22. 10.1111/j.1528-1157.1997.tb01242.x 9186255

[21] Lee W Low P Murugasu B Rajan U Epidemiology of epilepsy in Singapore children. Neurol J Southeast Asia (1997) 2:31–5.

[22] Mani K Rangan G Srinivas H Kalyanasundaram S Narendran S Reddy A. The Yelandur study: a community-based approach to epilepsy in rural South India—epidemiological aspects. Seizure (1998) 7:281–8. 10.1016/S1059-1311(98)80019-8 9733402

[23] Sridharan R Murthy B. Prevalence and pattern of epilepsy in India. Epilepsia (1999) 40:631–6. 10.1111/j.1528-1157.1999.tb05566.x 10386533

[24] Ng K Ng P Tsang K. Clinical characteristics of adult epilepsy patients in the 1997 Hong Kong epilepsy registry. Chin Med J. (2001) 114:84–7. 11779444

[25] Rajbhandari KC. Epilepsy in Nepal. Can J Neurol Sci. (2004) 31:257–60. 10.1017/S0317167100053919 15198454

[26] Tran D-S Odermatt P Le T-O Huc P Druet-Cabanac M Barennes H. Prevalence of epilepsy in a rural district of central Lao PDR. Neuroepidemiology (2006) 26:199–206. 10.1159/000092407 16569936

[27] Heaney DC MacDonald BK Everitt A Stevenson S Leonardi GS Wilkinson P. Socioeconomic variation in incidence of epilepsy: prospective community based study in south east England. BMJ (2002) 325:1013–6. 10.1136/bmj.325.7371.1013 12411362 PMC131020

[28] Faggiano F Partanen T Kogevinas M Boffetta P Socioeconomic differences in cancer incidence and mortality. IARC Sci Publ. (1996) (138):65–176. 9353664

[29] Guthmundsson K Hartharson P Sigvaldason H Sigfusson N. [Relationship between education and risk factors for coronary artery disease]. Nordisk Med. (1997) 112:169–75. 9273508

[30] Jones IR Urwin G Feldman RA Banatvala N. Social deprivation and bacterial meningitis in north east Thames region: three year study using small area statistics. BMJ (1997) 314:794. 10.1136/bmj.314.7083.794 9080999 PMC2126179

[31] Hemmingsson T Lundberg I Diderichsen F. The roles of social class of origin, achieved social class and intergenerational social mobility in explaining social-class inequalities in alcoholism among young men. Soc Sci Med. (1999) 49:1051–9. 10.1016/S0277-9536(99)00191-4 10475669

[32] Cubbin C LeClere FB Smith GS. Socioeconomic status and the occurrence of fatal and nonfatal injury in the United States. Am J Public Health (2000) 90:70. 10630140 10.2105/ajph.90.1.70 PMC1446109

[33] Vrijheid M Dolk H Stone D Abramsky L Alberman E Scott J. Socioeconomic inequalities in risk of congenital anomaly. Arch Dis Childhood (2000) 82:349–52. 10.1136/adc.82.5.349 10799420 PMC1718336

[34] Chang C-L Marmot MG Farley TM Poulter NR. The influence of economic development on the association between education and the risk of acute myocardial infarction and stroke. J Clin Epidemiol (2002) 55:741–7. 10.1016/S0895-4356(02)00413-4 12384187

[35] Karp A Kåreholt I Qiu C Bellander T Winblad B Fratiglioni L. Relation of education and occupation-based socioeconomic status to incident Alzheimer's disease. Am J Epidemiol. (2004) 159:175–83. 10.1093/aje/kwh018 14718220

[36] Luengo A Parra J Colas J Ramos F Carreras T Fernandez-Pozos M. Prevalence of epilepsy in northeast Madrid. J Neurol. (2001) 248:762–7. 10.1007/s004150170091 11596780

[37] Hovinga CA Asato MR Manjunath R Wheless JW Phelps SJ Sheth RD. Association of non-adherence to antiepileptic drugs and seizures, quality of life, and productivity: survey of patients with epilepsy and physicians. Epilepsy Behav. (2008) 13:316–22. 10.1016/j.yebeh.2008.03.009 18472303

[38] Thomson KE Modi AC Glauser TA Rausch JR Steve White H. The impact of nonadherence to antiseizure drugs on seizure outcomes in an animal model of epilepsy. Epilepsia (2017) 58:1054–62. 10.1111/epi.13742 28401980

[39] Dragoumi P Tzetzi O Vargiami E Pavlou E Krikonis K Kontopoulos E. Clinical course and seizure outcome of idiopathic childhood epilepsy: determinants of early and long-term prognosis. BMC Neurol. (2013) 13:206. 10.1186/1471-2377-13-206 24350775 PMC3878358

